# Predictive Value of Prognostic Nutritional Index on COVID-19 Severity

**DOI:** 10.3389/fnut.2020.582736

**Published:** 2021-01-14

**Authors:** Zhong-hua Wang, Ying-Wen Lin, Xue-biao Wei, Fei Li, Xiao-Long Liao, Hui-qing Yuan, Dao-zheng Huang, Tie-he Qin, Heng Geng, Shou-hong Wang

**Affiliations:** ^1^Department of Critical Care Medicine, Guangdong Provincial Geriatrics Institute, Guangdong Provincial People's Hospital, Guangdong Academy of Medical Sciences, Guangzhou, China; ^2^Department of Cardiology, Guangdong Cardiovascular Institute, Guangdong Provincial Key Laboratory of Coronary Heart Disease Prevention, Guangdong Provincial People's Hospital, Guangdong Academy of Medical Sciences, Guangzhou, China; ^3^Shantou University Medical College, Shantou, China; ^4^Emergency Department, The First People's Hospital of Jingzhou, Jingzhou, China; ^5^Department of Respiratory and Critical Care Medicine, The First People's Hospital of Shaoguan, Shaoguan, China; ^6^Department of Critical Care Medicine, The First People's Hospital of Jingzhou, Jingzhou, China

**Keywords:** prognostic nutritional index, COVID-19, severity, prognosis, nutrition – clinical

## Abstract

**Background:** The prognostic nutritional index (PNI) has been described as a simple risk-stratified tool for several diseases. We explored the predictive role of the PNI on coronavirus disease 2019 (COVID-19) severity.

**Methods:** A total of 101 patients with COVID-19 were included in this retrospective study from January 2020 to March 2020. They were divided into two groups according to COVID-19 severity: non-critical (*n* = 56) and critical (*n* = 45). The PNI was calculated upon hospital admission: 10 × serum albumin (g/dL) + 0.005 × total lymphocyte count (/mm^3^). Critical COVID-19 was defined as having one of the following features: respiratory failure necessitating mechanical ventilation; shock; organ dysfunction necessitating admission to the intensive care unit (ICU). The correlation between the PNI with COVID-19 severity was analyzed.

**Results:** The PNI was significantly lower in critically ill than that in non-critically ill patients (*P* < 0.001). The receiver operating characteristic curve indicated that the PNI was a good discrimination factor for identifying COVID-19 severity (*P* < 0.001). Multivariate logistic regression analysis showed the PNI to be an independent risk factor for critical illness due to COVID-19 (*P* = 0.002).

**Conclusions:** The PNI is a valuable biomarker that could be used to discriminate COVID-19 severity.

## Introduction

Coronavirus disease 2019 (COVID-19) is a life-threatening respiratory illness caused by severe acute respiratory syndrome coronavirus (SARS-CoV)-2 infection. On 11 March 2020, the World Health Organization (WHO) declared a pandemic due to COVID-19. SARS-CoV-2 is spreading rapidly worldwide and wreaking havoc in public healthcare systems. More than 65 million cases of SARS-CoV-2 infection have been recorded worldwide, including 1.5 million confirmed deaths up to December 2020, according to the WHO.

The clinical manifestations of COVID-19 are extremely broad. They range from asymptomatic disease and mild, self-limiting respiratory illness with nonspecific symptoms to severe pneumonia with life-threatening complications, including acute respiratory distress syndrome, multiorgan dysfunction, and death. These complications hamper appropriate care of these patients (1–4). It has been reported that 10–20% of patients diagnosed with COVID-19 require critical care, which places a huge burden upon healthcare facilities (5). Thus, early identification of patients at risk of becoming critically ill is vitally important to allocate medical resources and to provide early intervention to improve the prognosis. Interestingly, some epidemiological features and clinical characteristics have been identified as risk factors for COVID-19 severity (6). However, a novel, feasible, and readily assessable biomarker that can predict COVID-19 severity is not available.

The immune and nutritional status of the host is associated with susceptibility to and severity of infectious diseases (7–10). Hypoalbuminemia is present during inflammation and malnutrition, and is associated with poor clinical outcomes in acutely ill patients (11, 12). Conversely, white blood cell (WBC) counts in peripheral blood have been reported to reflect local or systemic inflammation (13). Lymphocytopenia is one of the most common clinical manifestations of acute viral infection (14, 15). Patients with severe COVID-19 exhibit prominent hypoalbuminemia and lymphocytopenia compared with those with non-severe COVID-19 (16–18). Analyses of molecular and immunological data from 326 patients with COVID-19 in Shanghai (China) revealed that host factors (especially lymphocytopenia) could predict disease progression (19).

The prognostic nutritional index (PNI) (20) is an objective assessment index reflecting the immune–nutritional status of patients. The PNI is calculated by multiplication of the serum albumin level and lymphocyte count. It is a valuable screening tool for patient prognosis in several diseases (21–24). Poor nutritional status and immune dysfunction (especially depletion of T lymphocytes) have been considered to be risk factors for severe infection by SARS-CoV-2 (25). However, the importance of this immune–nutritional index in terms of predicting COVID-19 severity has yet to be elucidated.

We explored the association of the PNI with COVID-19 severity and assessed its prognostic value using multivariate models.

## Materials and Methods

### Ethical Approval of the Study Protocol

The study protocol was approved by the Ethics Committee of The First People's Hospital of Jingzhou (L20200208) in Jingzhou, China. The requirement for written informed consent was waived due to the retrospective design of the study.

### Study Population

This observational retrospective study involved 101 patients with laboratory-confirmed SARS-CoV-2 infection at The First People's Hospital of Jingzhou (Jingzhou, China) from January 2020 to March 2020. A laboratory-confirmed case of COVID-19 was based on the result of real-time reverse transcription-quantitative polymerase chain reaction of a nasopharyngeal swab, in accordance with WHO guidelines (26). COVID-19 severity upon hospital admission was defined based on the fifth version of the *National Health Commission Guideline on the Management of Novel Coronavirus Pneumonia*. Patients were categorized into “critical” and “non-critical” COVID-19 groups. COVID-19 patients who were critically ill were identified if they had one of the following features: respiratory failure necessitating mechanical ventilation; shock; organ dysfunction necessitating admission to the intensive care unit (ICU).

### Data Collection

Demographic and clinical data were obtained retrospectively from electronic medical records in The First People's Hospital of Jingzhou, and assimilated by three researchers (Wang, Li, and Geng). Data were transferred to other team members in Guangdong Provincial People's Hospital (Guangzhou, China) for statistical analyses. The electronic medical records, nursing records, laboratory results, and imaging findings of all enrolled patients were reviewed. Data on age, sex, comorbidities, treatment plans, and duration of hospital stay were extracted. Laboratory results [complete blood count, hematology, coagulation testing, liver/renal function, as well as levels of electrolytes, C-reactive protein (CRP), procalcitonin, lactate dehydrogenase, and creatine kinase] were collected from the first electronic medical record after hospital admission. The PNI was calculated according to the following formula (20): PNI = 10 × serum albumin (g/dL) + 0.005 × peripheral lymphocyte count (/mm^3^). The Glasgow Prognostic Score (GPS) was obtained based on the level of CRP and albumin (27).

### Statistical Analyses

Statistical analyses were undertaken using SPSS 24.0 (IBM, Armonk, NY, USA). Continuous variables are expressed as the mean ± standard deviation (SD), median values, interquartile ranges or simple ranges. Categorical variables are summarized as counts and percentages. Continuous data with a normal distribution were compared using the Student's *t*-test. Data with a non-normal distribution were compared using the Wilcoxon rank-sum test and presented as the median and interquartile range. Categorical data were compared using the chi-square test or Fisher's exact test. Baseline data including demographic, history of illness, clinical and laboratory variables were included in the logistic analyses to assess their associations with COVID-19 severity. Univariate and multivariate logistic regression analyses were carried out to determine the risk factors for severe COVID-19. The PNI and variables with *P* < 0.05 in the univariate logistic regression analysis were included in the multivariate logistic regression analysis to evaluate COVID-19 severity. Then, the adjusted odds ratio (OR) and 95% confidence interval (CI) were calculated. The optimal cutoff of the PNI for predicting COVID-19 severity was determined by analyses of the receiver operating characteristic (ROC) curve. We also calculate the net reclassification improvement (NRI) and integrated discrimination improvement (IDI) to compare the discrimination ability between different prediction models. *P* < 0.05 was considered significant.

## Results

### Patient Characteristics at Baseline

A total of 101 patients with laboratory-confirmed COVID-19 were included in this study. Their demographic and clinical characteristics are listed in Table 1. The mean age of the study cohort was 56 ± 17 years. The study cohort comprised 47/101 (46.5%) males.

**Table 1:** Clinical characteristics stratified by the severity of COVID-19.

	**Non-critical (*n* = 56)**	**Critical (*n* = 45)**	***P*-value**
Age (years)	49.3 ± 16.9	64.9 ± 13.2	<0.001
**SEX**, ***N*** **(%)**			
Male	22 (39.3)	25 (55.6)	0.103
Female	34 (60.7)	20 (44.4)	
Hypertension, *n* (%)	13 (23.2)	15 (33.3)	0.259
Diabetes, *n* (%)	2 (3.6)	2 (4.4)	1.000
Heart rate, bpm	89.9 ± 15.3	90.0 ± 17.7	0.976
MAP, mmHg	97.0 ± 11.9	98.4 ± 19.3	0.693
CRP (mg/L)	7.4 (1.4, 24.5)	20.2 (7.2, 55.1)	0.002
SCr (μmol/L)	60.1 (51.0, 71.3)	73.9 (57.0, 86.2)	0.036
WBC count, × 10^9^/L	4.9 (3.7, 5.7)	6.1 (4.3, 8.6)	0.009
Neutrophil count, × 10^9^/L	3.1 (2.2, 3.9)	4.0 (2.6, 7.3)	0.004
Lymphocyte count, × 10^9^/L	1.3 (0.9, 1.6)	1.0 (0.6, 1.3)	0.005
Platelet count, × 10^9^/L	164.2 ± 68.1	172.0 ± 81.3	0.602
Hemoglobin (g/L)	121.8 ± 14.1	114.0 ± 21.9	0.041
**LIVER FUNCTION TESTS**			
ALT (U/L)	13.5 (9.3, 24.8)	26.0 (14.5, 48.0)	<0.001
Albumin (g/L)	41.4 ± 3.6	37.4 ± 4.3	<0.001
TBIL, μmol/L	11.2 (9.2, 13.3)	11.9 (9.4, 17.2)	0.367
DBIL, μmol/L	3.7 (3.0, 4.8)	4.5 (3.2, 6.4)	0.059
CK, U/L	63.0 (50.5, 88.5)	64.0 (32.5, 146.5)	0.874
CK–MB, U/L	12.0 (9.5, 15.0)	14.0 (9.3, 17.0)	0.252
PNI	48.0 ± 4.5	42.4 ± 5.4	<0.001
**TREATMENT**			
Antibiotic	55 (98.2)	31 (68.9)	<0.001
Glucocorticoid	22 (39.3)	29 (64.4)	0.012
Interferon	13 (23.2)	24 (53.3)	0.002
In-hospital death	0	6 (13.3)	0.017

*MAP, mean arterial pressure; CRP, C-reactive protein; SCr, serum creatinine; WBC, white blood cell; ALT, alanine transaminase; TBIL, total bilirubin; DBIL, direct bilirubin; CK, creatine kinase; CK-MB, creatine kinase-MB; PNI, prognostic nutritional index; MACEs, major adverse clinical events, defined by death, stroke, and requirement of dialysis*.

Among 101 patients with COVID-19, 45 (44.6%) were categorized into the critical group and 56 (55.4%) into the non-critical group. The mean age of the latter was 49.3 ± 16.9 years, whereas that of the critical group was 64.9 ± 13.2 years.

Patients critically ill with COVID-19 had a significantly higher risk of in-hospital death than those who were not critically ill with COVID-19 (13.3% vs. 0, *P* = 0.017). Compared with the non-critical group, patients in the critical group had a higher CRP level, higher WBC count, higher level of alanine aminotransferase (ALT), and lower hemoglobin level. Moreover, the PNI was significantly lower in the critical group as compared with that in the non-critical group (42.4 ± 5.4 vs. 48.0 ± 4.5, *P* < 0.001).

### Association of the PNI With COVID-19 Severity

Univariate logistic regression analysis showed that the PNI was associated with critical illness due to COVID-19 (odds ratio (OR) = 0.80, *P* < 0.001) (Table 2). The components of the PNI (serum level of albumin, and lymphocyte count) were strongly associated with critical illness due to COVID-19. Additional significant indicators were age, CRP level, estimated glomerular filtration rate <90 mL/min/1.73 m^2^, WBC count, anemia, and ALT level. After adjustment of these variables, multivariate regression analysis showed that the serum albumin level and lymphocyte count were independent predictors for critical illness due to COVID-19. When incorporated into multivariate analysis in model 2, the PNI remained an independent predictor for critical illness due to COVID-19 (OR = 0.81, 95%CI: 0.71–0.92, *P* = 0.002) (Table 3), indicating that the risk of critical COVID-19 would decrease by 19% with every unit increase of PNI. In addition, age was found to be independently associated with severe COVID-19 (OR 1.05, 95%CI: 1.01–1.10, *P* = 0.022).

**Table 2:** Univariate logistic regression analysis for the severity of COVID-19.

**Clinical variables**	**OR**	**95%CI**	***p*-value**
PNI	0.80	0.73, 0.88	<0.001
Age	1.07	1.04, 1.10	<0.001
Female sex	0.52	0.23, 1.15	0.105
Hypertension	1.65	0.69, 3.98	0.261
Diabetes	1.26	0.17, 9.28	0.823
Heart rate	1.00	0.98, 1.03	0.976
MAP	1.01	0.98, 1.03	0.676
CRP	1.02	1.00, 1.03	0.011
eGFR <90 ml/min/1.73 m^2^	3.94	1.37, 11.39	0.011
WBC count	1.21	1.06, 1.39	0.006
Lymphocyte count	0.29	0.12, 0.69	0.005
Platelet count	1.00	1.00, 1.01	0.598
Anemia	2.86	1.25, 6.51	0.013
Albumin	0.78	0.69, 0.88	<0.001
ALT	1.02	1.00, 1.03	0.029
TBIL	1.05	0.98, 1.13	0.158
DBIL	1.18	1.00, 1.39	0.052
CK	1.01	1.00, 1.01	0.069
CK–MB	1.02	0.98, 1.07	0.319

*OR, odds ratio; CI, confidence interval; PNI, prognostic nutritional index; MAP, mean arterial pressure; CRP, C-reactive protein; eGFR, estimated glomerular filtration rate; WBC, white blood cell; ALT, alanine transaminase; TBIL, total bilirubin; DBIL, direct bilirubin; CK, creatine kinase; CK-MB, creatine kinase-MB*.

**Table 3:** Multivariate logistic regression analysis for the severity of COVID-19.

**Clinical variables**	**OR**	**95%CI**	***p*-value**
**MODEL 1**			
Age	1.05	1.01, 1.10	0.019
CRP	0.99	0.97, 1.01	0.134
eGFR <90 ml/min/1.73 m^2^	2.36	0.54, 10.35	0.254
WBC count	1.34	1.01, 1.79	0.046
Anemia	1.04	0.30, 3.67	0.947
ALT	1.01	0.99, 1.03	0.33
Lymphocyte count	0.21	0.06, 0.73	0.014
Albumin	0.84	0.72, 0.98	0.022
**MODEL 2**			
Age	0.81	0.71, 0.92	0.002
CRP	0.99	0.97, 1.01	0.213
eGFR <90 ml/min/1.73 m^2^	2.46	0.57, 10.58	0.227
WBC count	1.26	0.98, 1.61	0.071
Anemia	0.98	0.29, 3.38	0.978
ALT	1.01	0.99, 1.03	0.195
PNI	0.81	0.71, 0.92	0.002

*OR, odds ratio; CI, confidence interval; CRP, C-reactive protein; eGFR, estimated glomerular filtration rate; WBC, white blood cell; ALT, alanine transaminase; PNI, prognostic nutritional index*.

### Comparison of the PNI and Its Components With Other Prognostic Factors

We explored the ability of the PNI and each of its components (serum level of albumin and lymphocyte count) using the area under the ROC curve (AUC) for the prediction of critical illness due to COVID-19.

**Figure 1:**
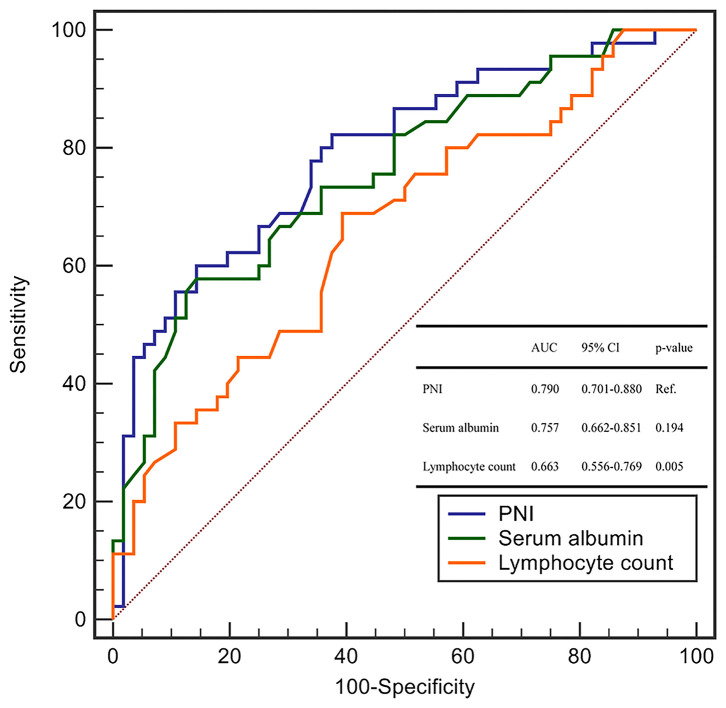
The ROC curves of PNI and its components for the prediction of critical COVID-19.

**Figure 2:**
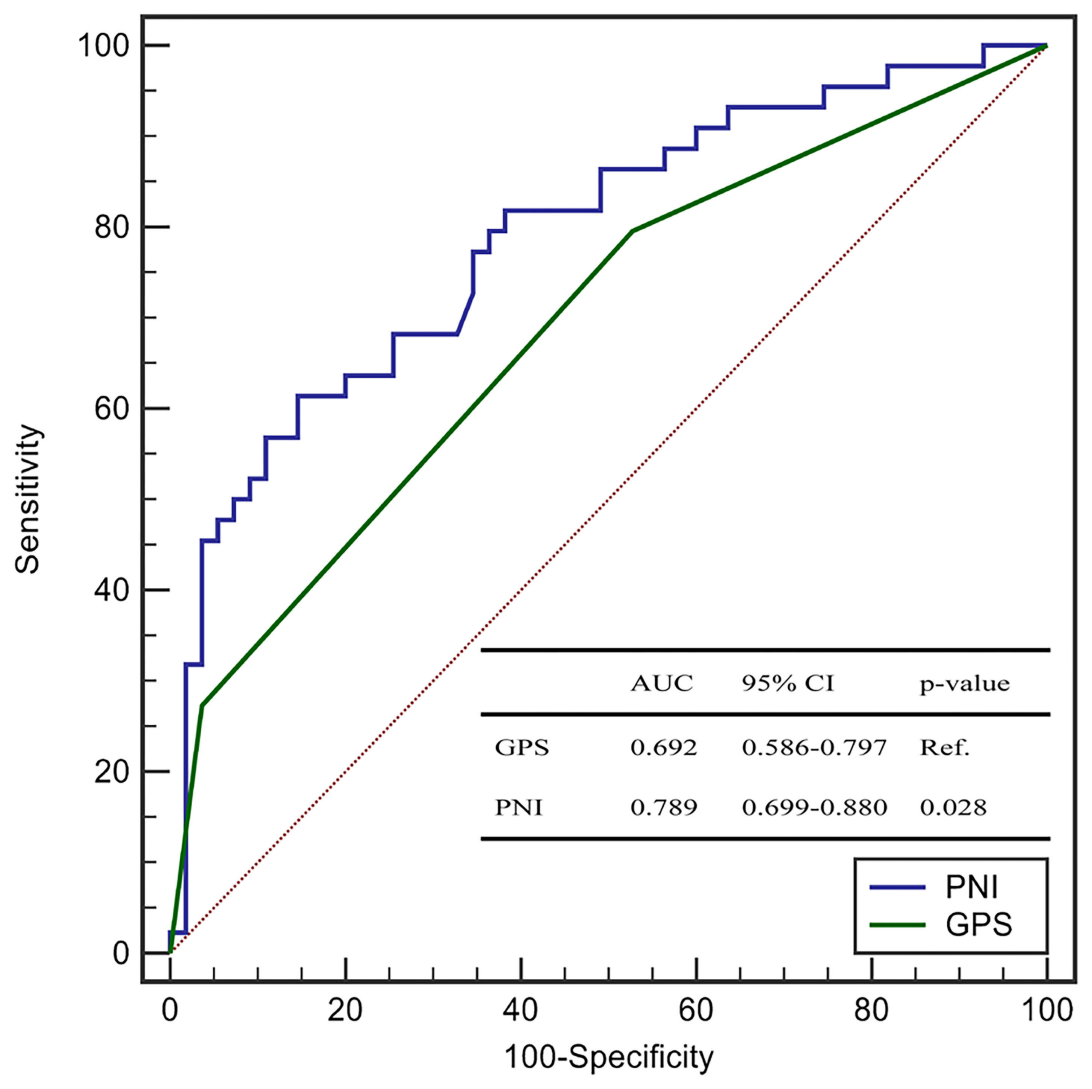
The ROC curves of PNI and GPS for the prediction of critical COVID-19.

The AUC of the PNI, serum level of albumin, and lymphocyte count was 0.790 (95%CI: 0.701–0.880), 0.757 (0.662–0.851), and 0.663 (0.556–0.769), respectively (Figure 1). The AUC of the PNI was significantly higher than that of the lymphocyte count but not the serum level of albumin. However, addition of the lymphocyte count to the serum level of albumin improved the prediction of COVID-19 severity (NRI = 0.4848, 95%CI: 0.1094–0.8604, *P* = 0.011; IDI = 0.0464, 95%CI: 0.0086–0.0844, *P* = 0.016). A PNI <43 was the optimal threshold for predicting severe COVID-19, had a sensitivity of 85.7% and specificity of 60.0%, and showed significantly higher accuracy than GPS for predicting critical illness due to COVID-19 (AUC: 0.789 vs. 0.692, *P* = 0.028) (Figure 2).

### PNI and Other Clinical Parameters

**Figure 3:**
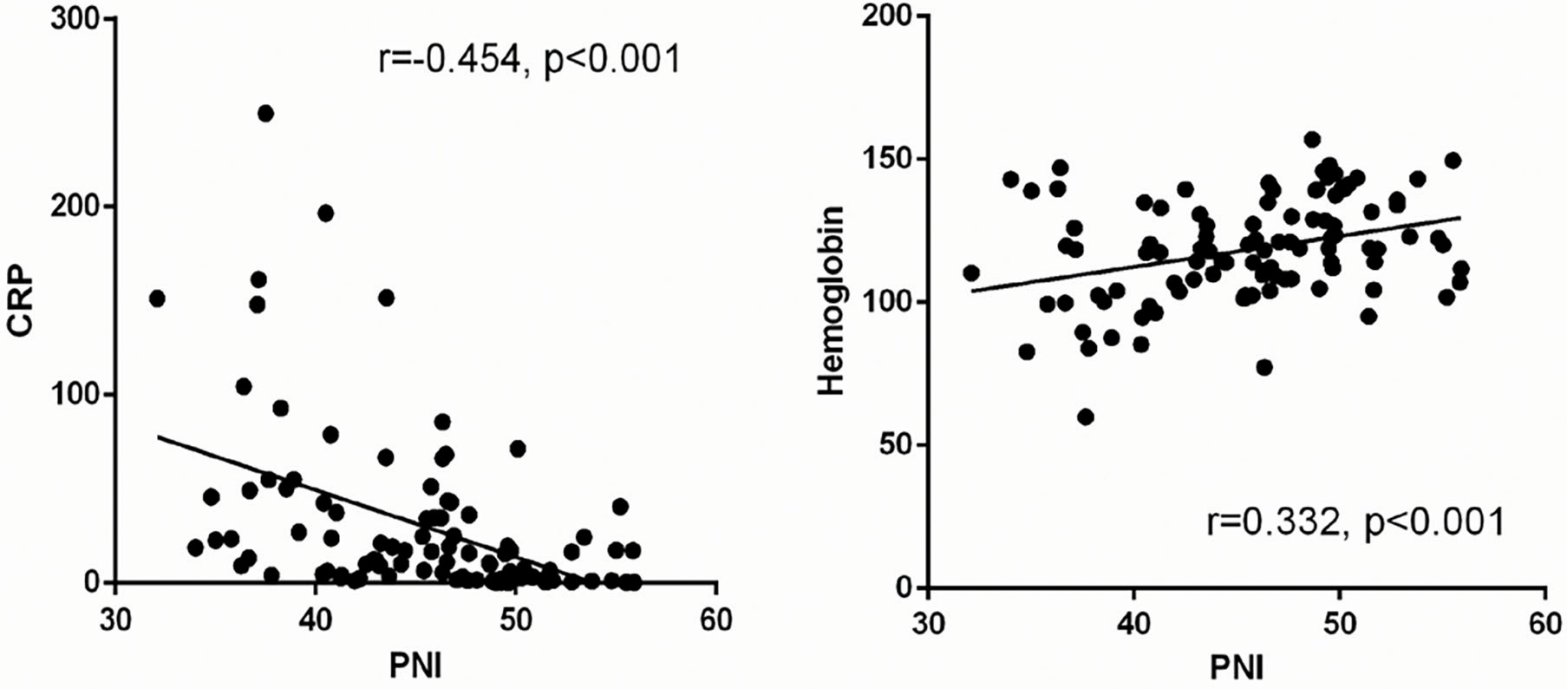
Linear regression between PNI and clinical parameters (CRP and hemoglobin).

The clinical characteristics of patients with COVID-19 stratified by the cutoff of the PNI level at the time of hospital admission are shown in Table 4. Patients with a PNI <43 were older with lower mean arterial pressure, higher level of CRP, higher level of creatine kinase-MB, and lower hemoglobin level. The prevalence of critical illness was significantly higher in patients with a lower PNI than that in patients with a higher PNI. Linear regression analysis showed that the PNI in patients with COVID-19 was negatively correlated with the CRP level and positively correlated with the hemoglobin level (*r* = −0.454, *P* < 0.001; *r* = 0.332, *P* < 0.001, respectively) (Figure 3).

**Table 4:** Clinical characteristics stratified by PNI level at admission.

	**PNI ≥ 43 (*n* = 69)**	**PNI < 43 (*n* = 32)**	***P*-value**
Age (years)	52.6 ± 17.4	64.3 ± 13.7	<0.001
**SEX**, ***N*** **(%)**			
Male	33 (47.8)	14 (43.8)	0.702
Female	36 (52.2)	18 (56.3)	
Hypertension, *n* (%)	17 (24.6)	11 (34.4)	0.309
Diabetes, *n* (%)	2 (2.9)	2 (6.3)	0.799
Heart rate, bpm	88.6 ± 15.0	93.0 ± 18.7	0.213
Mean arterial pressure, mmHg	100.7 ± 13.5	91.1 ± 17.8	0.003
C reactive protein (mg/L)	10.3 (1.5, 24.5)	25.4 (7.1, 73.1)	0.001
Serum creatinine (μmol/L)	63.4 (54.3, 80.4)	62.7 (48.6, 84.7)	0.506
White blood cell count, × 10^9^/L	5.1 (3.9, 6.6)	5.8 (4.1, 10.9)	0.116
Neutrophil count, × 10^9^/L	3.2 (2.3, 4.1)	4.6 (2.6, 9.3)	0.013
Lymphocyte count, × 10^9^/L	1.3 (1.0, 1.6)	0.8 (0.5, 1.0)	<0.001
Platelet count, × 10^9^/L	163.9 ± 71.1	175.7 ± 80.3	0.459
Hemoglobin (g/L)	122.6 ± 15.2	109.2 ± 21.2	0.002
**LIVER FUNCTION TESTS**			
ALT (U/L)	16.0 (11.0, 27.0)	26.5 (13.0, 66.5)	0.082
Albumin (g/L)	42.0 ± 2.7	34.6 ± 2.6	<0.001
TBIL, μmol/L	11.5 (9.5, 13.6)	11.9 (9.1, 18.5)	0.499
DBIL, μmol/L	3.9 (3.0, 4.7)	5.0 (3.2, 7.0)	0.049
Creatine kinase, U/L	67.0 (50.3, 97.0)	51.0 (31.3, 113.5)	0.241
Creatine kinase–MB, U/L	11.0 (9.0, 15.0)	15.0 (11.0, 22.0)	0.005
Critical cases, *n* (%)	20 (29.0)	25 (78.1)	<0.001
**TREATMENT**			
Antibiotic therapy	61 (88.4)	25 (78.1)	0.293
Glucocorticoid therapy	33 (47.8)	18 (56.3)	0.431
Interferon therapy	20 (29.0)	17 (53.1)	0.019
Length of stay, days	25 (20, 30)	26 (19, 35)	0.461
In-hospital death	2 (2.9)	4 (12.5)	0.148

*OR, odds ratio; CI, confidence interval; PNI, prognostic nutritional index; ALT, alanine transaminase; TBIL, total bilirubin; DBIL, direct bilirubin*.

## Discussion

The present study demonstrated the PNI to be an independent predictor of COVID-19 severity. A PNI <43 was the optimal cutoff for risk assessment. Risk factors (e.g., older age and PNI <43) could be used to identify potential critically-ill COVID-19 patients at an early stage. As compared with the GPS (which includes the CRP level and lymphocyte count), the PNI had significantly higher accuracy in predicting critical illness due to COVID-19.

The Chinese Center for Disease Control and Prevention reported that 81% of patients with COVID-19 have mild disease, 14% have severe disease, and 5% progress to critical illness with organ failure; thus, the mortality rate in the critically-ill group is ≤ 49% (28). Moreover, the interval from the initial development of symptoms to the onset of critical illness is 10 days (29). The delayed onset of critical illness in patients with COVID-19 and high mortality rate have prompted an urgent search for a biomarker that can allow an early diagnosis.

Herein, we found that older age was a risk factor for critical illness for COVID-19 patients, a finding that is consistent with the results of other studies (16, 30, 31). We also explored the predictive role of the PNI: it was independently associated with the severity of COVID-19 and a poor prognosis. The PNI is calculated based on the serum level of albumin and peripheral lymphocyte count. It was first conceptualized and applied by Buzby and colleagues to estimate the operative risk in gastrointestinal surgery (20).

Albumin is a well-known plasma protein with multiple physiological functions; the level of this protein fluctuates during inflammation and malnutrition (12, 32). Hypoalbuminemia has been strongly associated with poor clinical outcomes in acutely ill patients (11). Recent observational studies on patients with COVID-19 have demonstrated that a reduced albumin concentration in serum is associated with increased disease severity (17). Furthermore, the reduced production and increased loss of albumin in serum can be seen in patients with damage to liver function and renal function, which are common attributes in critically ill COVID-19 patients (33). In the current study, the PNI was negatively correlated with the CRP level. Interestingly, an inflammatory status promotes albumin degradation and a reduction in its level in the liver (34, 35); severe inflammation is associated with a progressively lower serum albumin level (34). Accumulating evidence has suggested that a subgroup of patients with severe COVID-19 exhibit a hyperinflammatory status with a “cytokine storm,” which further supports the potential predictive role of hypoalbuminemia (2, 36, 37).

Lymphocytopenia has been shown to be associated with increased severity of COVID-19 (38). Patients who died from COVID-19 were reported to have a significantly lower lymphocyte count than that of survivors (33, 37). A dramatically reduced number of cluster of differentiation (CD)4+ T cells, CD8+ T cells, B cells, and natural killer (NK) cells has been noted in patients with severe COVID-19 (17, 39). Expression of NKG2A (a marker of the exhausted function of NK cells and CD8+ T cells) is increased significantly in COVID-19 patients (40). In addition, restoration of the lymphocyte count from peripheral blood has been seen in patients with viral clearance (41). The potential compounding immunological insults caused by SARS-CoV-2 are major mechanisms of COVID-19 progression, which suggests that surveillance of the lymphocyte count is valuable in the early screening of critical illness due to COVID-19 (42). Intriguingly, treatments that address the immunopathology of SARS-CoV-2 are under intensive focus currently (43).

Taken together, it can be deduced that the PNI characterizes the immune and inflammatory status in patients with COVID-19. We validated, for the first time, the PNI as a useful biomarker that is independently associated with COVID-19 severity. We also compared the discrimination ability of the PNI with its components. The PNI conferred a significantly higher predictive value for COVID-19 severity than that by the lymphocyte count. However, when compared with the serum level of albumin, the PNI had a higher AUC for prediction of COVID-19 severity but was not significant using the Concordance Index (C-Index). However, analyses of the NRI and IDI demonstrated that the PNI (which contains the albumin level and lymphocyte count) conferred additional performance for the prediction of critical illness due to COVID-19 as compared with that of albumin level alone. Although the C-Index and AUC are the most popular metrics for evaluating the discrimination performance of prognostic models, both have limitations. Once the C-Index or AUC reach a certain level, they require large effect sizes from the newly added markers to obtain a noticeable increase, but such large effect sizes are uncommon and unrealistic. The NRI is a simple (but effective) approach for comparing the discrimination ability between two models, especially for the improvement conferred by inclusion of new variables (44).

The present study had three main limitations. First, nearly half of enrolled patients in the study had critical illness due to COVID-19. This high proportion of critically ill patients in the study cohort could have been because our hospital was the main facility in Jingzhou responsible for admitting and caring for critically ill patients with COVID-19. Second, due to the retrospective design and the urgency of the epidemics, anthropometrics data is lacking and factors such as levels of interleukin-6 and ferritin were not measured, so their role in predicting COVID-19 severity could not be considered during data analyses. Third, this was a single-center study with a small study cohort and small number of critically ill cases, which would have underpowered our analyses.

## Conclusions

The PNI is a valuable and inexpensive biomarker that is independently associated with COVID-19 severity. Patients with a PNI <43 were likely to be critically ill with COVID-19. Our findings could help clinicians to identify patients at a risk of critical illness at an early stage of COVID-19 and who have a poor prognosis.

## Data Availability Statement

The original contributions presented in the study are included in the article/supplementary material, further inquiries can be directed to the corresponding author/s.

## Ethics Statement

The studies involving human participants were reviewed and approved by the Ethics Committee of The First People's Hospital of Jingzhou (L20200208). Written informed consent for participation was not required for this study in accordance with the national legislation and the institutional requirements.

## Author Contributions

S-hW and HG contributed to the conception or design of the study. Z-hW, FL, Y-WL, X-bW, X-LL, H-qY, and D-zH contributed to the acquisition, analyses, or interpretation of data. Z-hW, Y-WL, and X-bW drafted the manuscript. S-hW and T-hQ revised the manuscript critically. All authors agree to be accountable for all aspects of work to ensure its integrity and accuracy. All authors contributed to the article and approved the submitted version.

## Conflict of Interest

The authors declare that the research was conducted in the absence of any commercial or financial relationships that could be construed as a potential conflict of interest.
